# The role of EGFR double minutes in modulating the response of malignant gliomas to radiotherapy

**DOI:** 10.18632/oncotarget.20714

**Published:** 2017-09-08

**Authors:** Yi-Hong Zhou, Yumay Chen, Yuanjie Hu, Liping Yu, Katherine Tran, Erich Giedzinski, Ning Ru, Alex Gau, Francine Pan, Jiao Qiao, Naomi Atkin, Khang Chi Ly, Nathan Lee, Eric R. Siegel, Mark E. Linskey, Ping Wang, Charles Limoli

**Affiliations:** ^1^ UC Irvine Brain Tumor Laboratory and Department of Surgery, University of California Irvine, Irvine, CA, USA; ^2^ UC Irvine Diabetes Center and Department of Medicine, University of California Irvine, Irvine, CA, USA; ^3^ Department of Radiation Oncology, University of California Irvine, Irvine, CA, USA; ^4^ Departments of Biostatistics, University of Arkansas for Medical Sciences, Little Rock, AR, USA

**Keywords:** glioblastoma multiforme, EGFR, double minute (DM) chromosome mis-segregation, radiation, tumor heterogeneity

## Abstract

*EGFR* amplification in cells having double minute chromosomes (DM) is commonly found in glioblastoma multiforme (GBM); however, how much it contributes to the current failure to treat GBM successfully is unknown. We studied two syngeneic primary cultures derived from a GBM with and without cells carrying DM, for their differential molecular and metabolic profiles, *in vivo* growth patterns, and responses to irradiation (IR). Each cell line has a distinct molecular profile consistent with an invasive “go” (with DM) or angiogenic “grow” phenotype (without DM) demonstrated *in vitro* and in intracranial xenograft models. Cells with DM were relatively radio-resistant and used higher glycolytic respiration and lower oxidative phosphorylation in comparison to cells without them. The DM-containing cell was able to restore tumor heterogeneity by mis-segregation of the DM-chromosomes, giving rise to cell subpopulations without them. As a response to IR, DM-containing cells switched their respiration from glycolic metabolism to oxidative phosphorylation and shifted molecular profiles towards that of cells without DM. Irradiated cells with DM showed the capacity to alter their extracellular microenvironment to not only promote invasiveness of the surrounding cells, regardless of DM status, but also to create a pro-angiogenic tumor microenvironment. IR of cells without DM was found primarily to increase extracellular MMP2 activity. Overall, our data suggest that the DM-containing cells of GBM are responsible for tumor recurrence due to their high invasiveness and radio-resistance and the mis-segregation of their DM chromosomes, to give rise to fast-growing cells lacking DM chromosomes.

## INTRODUCTION

Glioblastoma multiforme (GBM) is one of the most aggressive forms of brain cancer and characteristically recurs despite treatment with post-operative radiation and chemotherapy. Overall survival of patients has been shown to depend on the specific molecular GBM subtype, with proneural and neural subtypes showing poorest response to conventional therapeutic intervention, while classical and mesenchymal subtypes exhibit statistically significant, albeit quantitatively marginal, improvements in survival through extensive tumor resection and chemo/radiation therapies [1]. Intra-tumoral heterogeneity is a hallmark of GBM where the “M” stands for “multiforme”, and where subpopulations of cells can be distinguished based on their phenotypic resemblance to neuronal-glial stem cells. Subpopulations that exhibit properties similar to neuronal-glial stem cells and tumor-initiating capabilities, referred to as stem-like tumor initiating cells (STIC), differ from the majority of cells forming the tumor mass, referred to as tumor mass-forming cells (TMC). Each tumor subpopulation possesses different genetic and/or epigenetic abnormalities and has molecular phenotypes that impact a variety of tumorigenic behaviors. In GBM, STIC were shown to participate directly in tumor vascularization [2, 3].

Significant evidence points to the intra-tumoral heterogeneity of GBM as a major confounding factor for therapy, as plasticity of the subpopulation phenotypes, driven by evolving selective pressures within the tumor microenvironment, promote treatment resistance and tumor recurrence. This is supported by much of our past work demonstrating that clinically relevant irradiation (IR) paradigms were associated with increased oxidative stress, radio-resistance, cellular reprogramming and invasive behavior, of which the latter could be mediated by radiation-induced macrovesicles [4, 5]. Similarly, work by ourselves and others has also substantiated the importance of *EGFR* amplification in GBM, either as chromosomal 7 polysomy or as double minute chromosomes (DMs) [6, 7]. However, *EGFR* amplification was not predictive of survival in glioma treated with an inhibitor of EGFR concurrently with chemo and radiation therapy [8], and supporting evidence is limited for the role of *EGFR*-encoding DM in GBM recurrence following frontline radiotherapy.

To further understand the mechanisms of GBM recurrence and the role that *EGFR* amplification plays in this process, we conducted studies using two syngeneic primary cultures of a likely classical GBM subtype. Each of these carefully characterized primary cultures expresses wild-type/functional proteins of TP53, PTEN, IDH1, and IDH2, but differed in regard to the presence (cell line 51A) or absence (cell line 51B) of DM, and gene/protein expression patterns. Here we provide evidence in support of a GBM tumor recurrence mechanism dependent on tumor heterogeneity and favored by tumor-specific chromosomes, herein EGFR-encoding DMs.

## RESULTS

### Characterization of EGFR status in syngeneic primary cultures of a GBM

**Figure 1:**
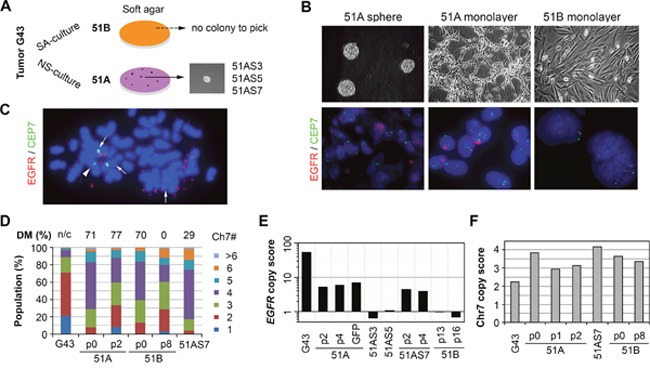
NS-culture conditions maintained and enriched the GBM cell subpopulation carrying EGFR-encoding double minute chromosomes (DM) **(A)** depiction of the establishment of 51A and 51B primary cultures and their clonal lines expanded from single colonies formed in 0.3% soft agar. **(B)** representative microscope images of cell cultures (upper) and FISH (bottom). **(C)** a metaphase image of FISH of 51A showing near diploid nucleus with nearly forty copies of the EGFR double minute chromosomes, EGFR-encoding Chr7 is shown by an *arrow*, and the EGFR-deleted Chr7 by an *arrowhead*. **(D)** Chr7-copy number defined cell subpopulation equilibrium in the G43 tumor and its derived cell lines after various numbers of cell passages (p). The data were calculated based on more than 250 cells, with percentage of DM cells shown above the bar for the Chr7-cell populations. DM cells are shown in FISH images of the G43 tumor, but not counted (n/c). **(E)** *EGFR* gene copy score computed as ratios between *EGFR* and reference gene *SPAG16* DNA copy numbers, determined by real-time comparative quantification (CQ-) PCR and normalized to human leukocyte DNA, which was set to unity. **(F)** Chr7 copy score computed based on the average number of FISH-detected copies per cell.

The primary human cultures established under neural sphere (NS) or serum adherent (SA) culture conditions, as depicted in Figure 1A, were derived from a recurrent GBM patient (G43) 14 months after initial resection followed by Temozolomide and radiotherapy (standard of care), and Avastin and Velcade (second line salvage therapies). Mutation assays showed wild-type sequences for *PTEN, TP53, IDH1* and *IDH2* genes in the original tumor and the two syngeneic primary cultures (51A and 51B).

Analysis of G43 DNA by comparative quantitative (CQ-) PCR indicated a 54-fold higher amplification of the *EGFR* gene compared to a standard reference gene [6]. As shown in Figure 1B-1C, fluorescence *in situ* hybridization (FISH) revealed EGFR-bearing DMs in cells that were maintained and slightly enriched (from 71% to 77%) in NS-culture; however, these were completely lost in SA-culture after 8 passages (Figure 1D). CQ-PCR also showed copy number variation of *EGFR*, normalized to the reference gene *SPAG16*, in the original tumor and derived cultures at various passages (Figure 1E). In contrast to similar variations of Chr7 (Figure 1D and 1F), *EGFR* copy score showed a high level of *EGFR* gene amplification in 51A and its clonal line 51AS7, but not in 51B or the other two clonal lines of 51A (51AS3 and 51AS5), which originated from soft-agar colonies (Figure 1A). A decrease in *EGFR* copy score was seen in 51AS3 and 51B after many passages. This may be associated with a decrease of chromosome 7 score, which was observed directly in 51B (Figure 1F).

### Restoration of tumor heterogeneity by mis-segregations of Chr7 and DM

In the procedure of soft-agar-mediated cloning (Figure 1A), clonal cell lines were established from 51A (51AS3, 51AS5, 51AS7), but not from 51B. Both FISH and CQ-PCR consistently showed an *EGFR*-DM cell origin of 51AS7; however, the percentage (29%) of cells with DM was much lower than that in the parental culture (77%). In contrast, the equilibrium of tumor heterogeneity of Chr7, and Chr7 copy score, were similar between the clonal line (51AS7) and two initial primary cultures (51A p0 and 51B p0) (Figure 1D, 1F). Hence, DM cells were able to re-colonize and restore tumor heterogeneity by mis-segregations of Chr7 and DM, and DM-heterogeneity was likely related to tumor-specific phenotypes, as proven below, by comparative analyses of two GBM syngeneic primary cultures, with and without cells carrying DM.

### Molecular profiles of cells with or without DM

**Figure 2:**
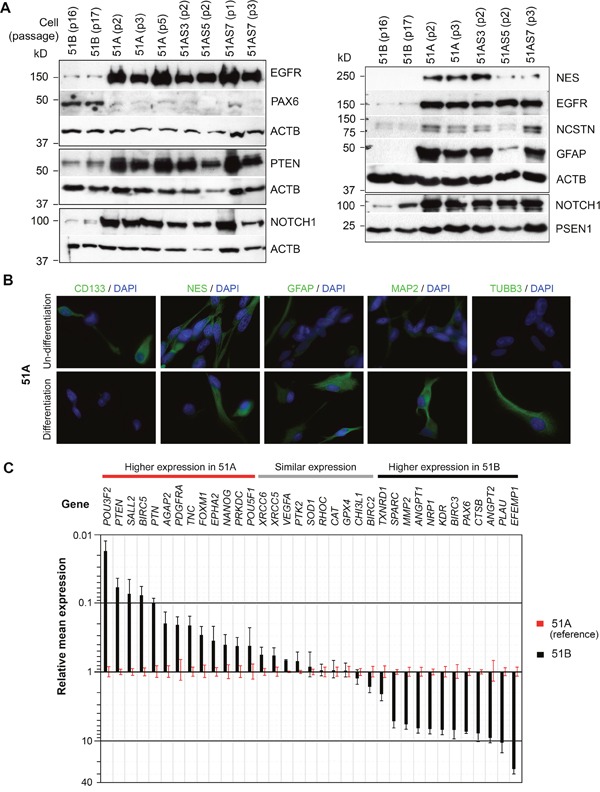
Differential molecular profiles and phenotypic behaviors of GBM cell subpopulations with and without DM **(A)** immunoblotting whole cell lysates of primary cultures (51A and 51B) and clonal lines of 51A (51AS3, 51AS5, and 51AS7). Cell passages are shown within parentheses. **(B)** immunocytofluorescence analysis of monolayer cultures of 51A before and after a 7-day culture under neural stem cell differentiation conditions. **(C)** comparison of gene expressions between 51A and 51B, quantified by real-time qRT-PCR, normalized to *ACTB.* Error bars represent SEM derived from 3 replicates.

A primary culture (51A) and clonal derivatives with or without DM (51AS3, 51AS5, 51AS7) cultured under NS conditions exhibit equivalent high level of full-length (175 kD) EGFR, as determined by immunoblotting analysis. In contrast, a primary culture (51B) maintained under SA-conditions demonstrated consistently low levels of EGFR (Figure 2A). Cells cultured under both NS and SA-conditions expressed wild-type (55 kD) PTEN, with NS-cultures (51A, 51AS3, 51AS5, 51AS7) exhibiting significantly higher PTEN expression than the SA-culture (51B). In addition, all EGFR-overexpressing NS-cultures (except 51AS5) showed high levels of NOTCH1 and γ-secretases (NCSTN and PSEN1) of NOTCH signaling (Figure 2A, right panel).

A high level of proteins in the NOTCH signaling pathway and GFAP was commonly seen in both neuronal-glial stem/precursor and glioma stem-like cells [9], which is consistent with the neural stem cell phenotype of cells expected for NS-cultures of GBM. On the other hand, SA-cultures of 51B showed high expression of the early neuronal differentiation marker PAX6, which was rarely expressed by any of the NS-cultures (Figure 2A, left panel). Hence, there were two principal protein profiles that characterized the two key cell subpopulations of this GBM; PAX6^low^/EGFR-PTEN-NOTCH^high^ (51A, NS-culture), and PAX6^high^/EGFR-PTEN-NOTCH^low^ (51B, SA-culture). All clonal NS-subculture lines with (51AS7) or without (51AS3 and 51AS5) DM showed the same protein profile of PAX6^low^/EGFR-PTEN-NOTCH^high^ as their parental NS-culture line 51A with DM.

We then used a neural stem cell differentiation assay to verify the neural stem cell feature of 51A suggested by its neurosphere phenotype and neural stem-cell marker expression. As shown in Figure 2B, immunocytofluorescence analysis showed expression of CD133, a common marker of glioma stem cells, by 51A under non-differentiation culture conditions, which was diminished following 1-week of culture under differentiation conditions. Expressions of neuronal markers (MAP2 and TUBB3), which had low or undetectable expression prior to differentiation were increased/detected following differentiation. Decrease of CD133 and increase of MAP2 and TUBB3 expressions under differentiation conditions are characteristic features of neuronal precursor cells. Different from neuronal precursor, 51A expressed both NES and GFAP under both non-differentiation and differentiation conditions.

**Figure 3:**
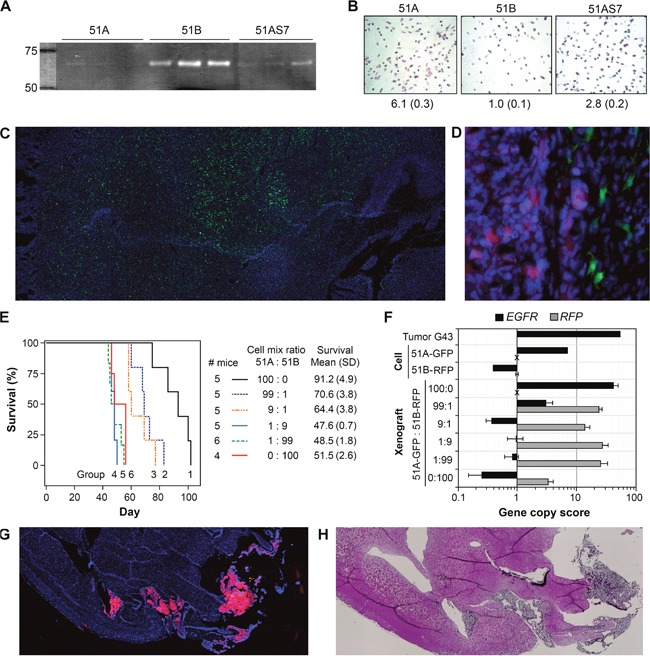
Distinct “go” or “grow” features of STIC and TMC from GBM with or without EGFR amplification **(A)** gelatin zymography of protein precipitated from 48-hour conditioned medium of cell cultures in triplicate. **(B)** HEMA staining of cells in a matrigel invasion assay. The relative invasion rates (SD) are shown below the representative images of invading cells, with 51B cell invasion rate set to unity. **(C)** representative fluorescence image of a mouse brain with i.c. xenografts derived from 51A-GFP. **(D)** a peritumoral region of i.c. xenograft derived from co-implantation of 51A-GFP and 51B-RFP in a 9:1 ratio. **(E)** Kaplan-Meier survival curves of mice with i.c. implantation of 51A infected with a lentiviral vector expressing green fluorescence protein (51A-GFP) and 51B infected with a lentiviral vector expressing red fluorescence protein (51B-RFP) alone and in combinations at various ratios in six groups (5 mice per group). **(F)** CQ-PCR analysis of DNA samples from cells and the right halves of brains of euthanized mice. **(G** and **H)** fluorescence and H&E images of a mouse brain with i.c. xenografts derived from 51B-RFP. Pictures were taken using 10X (C, G, and H) or 40X (D) lens of a Keyence BZ-X700 fluorescence microscope with multiple images seemliness stitched into one.

In order to delineate the different roles of tumor cell subpopulations in tumor recurrence after radiation, we applied the absolute quantitative reverse transcription PCR (AqRT-PCR) to measure the absolute ratio of a marker gene to an internal control gene (here *ACTB*) [10] and compared their differential expression in 51A and 51B. Marker genes were selected that encoded proteins with functions in early central nervous system development (NANOG, POU3F2, POU5F1, PAX6, PTEN, PDGFRA, and SALL2), matrix re-modeling, cell mobility and invasion (EFEMP1, AGAP2, CTSB, EPHA2, FOXM1, MMP2, MMP9, PLAU, PTK2, RHOC, SPARC, and TNC), tumor vascularization (ANGPT1, ANGPT2, FLIT1, NRP1, KDR, VEGFA, and PTN), DNA repair and cell survival (BIRC2, BIRC3, BIRC5, PRKDC, TXNRD1, XRCC5, and XRCC6), redox metabolism (CAT, GPX4, and SOD1), and glioma radioresistance (CHI3L1). As shown in Figure 2C, consistent with NS-culture-derived glioma cells with high invasiveness (Figure 3), genes highly expressed in 51A were those encoding markers of neural stem/precursor cells (NANOG, POU3F2, POU5F1, and SALL2), and pro-invasive proteins (AGAP2, EPHA2, FOXM1, PDGFRA, and TNC). In contrast, genes encoding angiogenic factors (ANGPT1, ANGPT2) and receptors (NRP1, and KDR) were highly expressed in SA-culture-derived glioma cells of 51B. Expression of *MMP9* and *FLIT1* was not detected, and expression of the angiogenic factor *VEGFA* was not differentially expressed between the two syngeneic glioma primary cultures.

### “Go” or “grow” functional dependence on DM

Both AqRT-PCR and gelatin zymography assays confirmed the absence of MMP9 in 51A and 51B, but MMP2 was highly expressed, secreted, and activated in the extracellular compartment of 51B, in comparison to that of 51A (Figure 3A). We then examined tumor cell invasiveness by a matrigel invasion assay that was used to show MMP2-dependent invasion of glioma cell line U251 [11]. As shown in Figure 3B, the high level of active MMP2 in the extracellular compartment of 51B did not translate into high cell invasiveness, as compared to 51A and 51AS7, despite high expression of genes (*CTSB, EFEMP1, PLAU*, and *SPARC*) with pro-invasive properties. However, the level of invasiveness was positively related to the percentage of DM cells, which may be due to enhanced expression of another set of pro-invasive genes, including those (*AGAP2, PDGFRA, TNC*, and *FOXM1*) in 51A, as shown in Figure 2C.

To examine the impact of the different phenotypes of 51A and 51B cells, suggested by their distinct molecular profiles, on their roles in tumor formation, we carried out intracranial (i.c.) implantation of defined mixtures of 51A and 51B, each having previously been transduced with lentiviral vectors to express green (GFP) or red (RFP) fluorescence proteins, respectively. Keeping the total implanted cell number constant, the tumorigenicity of 51A-GFP and 51B-RFP cells was followed after implanting each line alone or in various proportions into the right hemispheres of nude mouse brains. All mice were followed to ethical end-points and their brain tissues were cryo-sectioned and observed under a fluorescence microscope following nuclear staining with DAPI.

Consistent with the result of the matrigel invasion assay, 51A cells were highly invasive *in vivo*. As shown in Figure 3C, a diffused tumor cell distribution throughout the mouse brain was observed in xenografts derived from i.c. implantation of 51A-GFP cells. In contrast, 51B-RFP cells formed large clusters of tumor masses, even when started as the minority (10%) of cells mixed with a majority (90%) of 51A-GFP cells, indicating the tumor-mass-forming feature of 51B cells (Figure 3D). In xenografts derived from such proportion of mixing, the percentage of 51A-GFP cells was calculated using the software provided with a Keyence BZ-X700 fluorescence microscope, which captured areas of green signals within the tumor mass and areas of green signals outside the tumor mass, which was readily marked by the red fluorescence signal from 51B-FRP cells. The counts and size (μm^2^) of the total green areas vs the total green inside the tumor mass are 77 and 13403 vs 43 and 10211, with area averages (SD) as 174 (374) vs 237 (481), respectively. Based on count, 44% 51A-GFP cells were outside the tumor mass, while based on area, it was 24%. The greater variations on area average but larger overall area within the tumor mass compared to those of the total green areas suggest a smaller and more uniform morphology of 51A-GFP cells outside the tumor mass.

Consistently, mice implanted with a majority (100%, 99% or 90%) of 51A cells (groups 1-3) versus a majority (100%, 99% or 90%) of 51B cells (groups 4-6) showed significantly longer survival as revealed by Kaplan-Meier curves. In other words, adding as little as 1% of 51B cells to 51A cells in the inoculum significantly shortened the survival time of mice (*P* < 0.001 in the comparison between groups 1 and 2) (Figure 3E). Thus, the highly invasive 51A cells had a lower level of i.c. tumorigenicity than the 51B cells, the latter having an angiogenic molecular profile *in vitro* (Figure 2B) and a local proliferation phenotype *in vivo* (Figure 3D).

To determine the differential growth speed of each cell subpopulation *in vivo*, we extracted DNA from the right hemisphere of each mouse's brain in which the pre-mixed 51A-GFP and 51B-RFP cells had been implanted and developed into a tumor, and performed CQ-PCR to quantify the copy numbers of *GFP, RFP, EGFR* and *SPAG16* on DNA of 3-4 mice per group. As shown Figure 3F, the level of *EGFR* amplification in the 51A-derived xenograft was similar to that of its parental clinical tumor, confirming the STIC nature of EGFR-DM containing cells. The DNA encoding GFP can be detected by CQ-PCR in 51A-GFP derived xenografts, and its frequency is comparable to *SPAG16* (ratio = 0.7). In xenografts derived from the mixture of 99% 51A-GFP and 1% 51B-RFP, the ratio was dramatically decreased to 0.1, and was undetectable in xenografts from mixtures with a higher proportion of 51B-RFP cells. This demonstrated the fast-growing phenotype of 51B cells *in vivo* with quantitative data, as shown by images of the xenografts (Figure 3D). Interestingly, we observed an increase of RFP copy number in 51B-RFP cells in forming the tumor (xenograft) compared to its copy number for *in vitro* cell culture. These data suggest that the proliferative potential of cells grown *in vivo* can be distinct from *in vitro* measurements, and that these differential effects can have a significant impact on tumorigenicity derived from implanting defined mixtures of cells. Both immunofluorescence and H&E images of a mouse brain with i.c. xenografts derived from 51B-RFP showed the fast proliferating characteristics of 51B cells, with solid tumor masses shown in the brain (Figures 3G-3H), which was in striking contrast to the diffused tumor cell distribution throughout the mouse brain of i.c. xenografts derived from 51A-GFP (Figure 3C). The decreased survival of mice with i.c. xenografts derived from 51A100% and mixture of 51A 99% and 51B 1% can be explained by these mice having their brains damaged from the massive growth of solid tumors, which were contributed by the tumor mass-forming cells of 51B, as shown in Figures 3D and 3F.

### Differential cellular bioenergetics and radiation resistance

**Figure 4:**
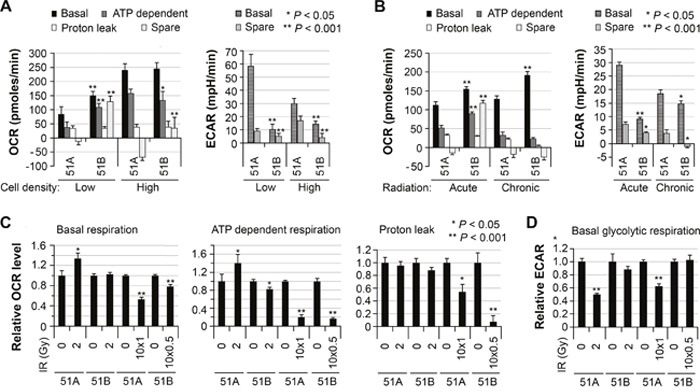
Differential cellular bioenergetics of two GBM subpopulations that changed in response to radiation **(A)** comparison of basal oxygen consumption rate (OCR) and extracellular acidification (ECAR) levels for 51A and 51B using an extracellular flux analyzer in two independent experiments. The cells were cultured for 7 days from 20,000 cells per well (left panel) and for 3 days from 140,000 cells per well (right panel) in XF24-plates. 51A and 51B cells were monolayer cultures throughout the experiments, in each specified culture medium. Oligomycin (a complex V inhibitor), FCCP (an uncoupler), and Rotenone (a complex I inhibitor) were injected sequentially to assay the mitochondrial respiration capacity. The basal respiration, ATP-linked respiration (oligomycin-sensitive fraction), proton leak (oligomycin-insensitive fraction), mitochondrial reserve (spared respiration), basal glycolytic respiration and reserved glycolytic respiration are compared. Error bars correspond to the SEM of measurements. **(B)** comparison of OCAR and ECAR levels between irradiated 51A and 51B cells, 7 or 11 days post-acute (single dose of 2Gy, low cell density) or chronic (1Gy to 51A or 0.5 Gy to 51B per day for 10 consecutive days, high cell density) IR, respectively. **(C** and **D)** comparison of OCAR and ECAR levels in irradiated and un-irradiated cells, with the latter set to unity.

The above characterization of GBM subcultures with *EGFR* amplification showed the presence of stem-like invasive cells with DM in 51A and fast-growing cells without DM in 51B. We then further studied their metabolic parameters by subjecting both primary cultures to extracellular flux analyses. In exponentially growing cultures (low confluence), both basal and ATP-linked (oligomycin-sensitive fraction) oxidative phosphorylation was significantly higher in 51B than in 51A (Figure 4A, left panel). Under slower growth conditions (high confluence), basal and ATP-dependent respiration increased in both lines, with no significant differences between the two lines (Figure 4A, left panel). There was no difference in OCR from proton leakage between the two lines at both low and high cell confluence (Figure 4A, left panel). Furthermore, reserve respiration capacity could not be detected in 51A (the 51A cells were very sensitive to FCCP, especially at high density, and resulted in the reduced OCR), suggesting that the ATP-reserve was exhausted. This was in contrast to 51B, where exponentially growing cells exhibited significant mitochondrial reserve that dissipated as cultures reached confluence. However, glycolytic respiration shown by ECAR was significantly higher in 51A versus 51B, and was not dependent on culture confluence (Figure 4A, right panel).

Similar studies were then conducted to determine the impact of acute (2 Gy) or chronic (10 × 0.5 Gy or 10 × 1.0 Gy) IR on cellular bioenergetics 7 or 11 days following exposure, respectively. Following IR, glycolytic respiration and oxidative phosphorylation levels remained higher and lower, respectively, in 51A compared to 51B (Figure 4B), as was the case prior to radiation (Figure 4A). Further analysis showed that 1 week after 2-Gy radiation, basal and ATP-dependent mitochondrial respiration was increased and basal glycolytic respiration significantly decreased in 51A cells, while no changes were found in 51B cells (Figure 4C-4D). However, chronic IR compromised both basal and ATP-dependent mitochondrial respiration with decreased proton flux in both 51A and 51B cultures (Figure 4C). Both acute and chronic radiation of 51A caused a reduction of glycolytic respiration, but not for 51B (Figure 4D).

**Figure 5:**
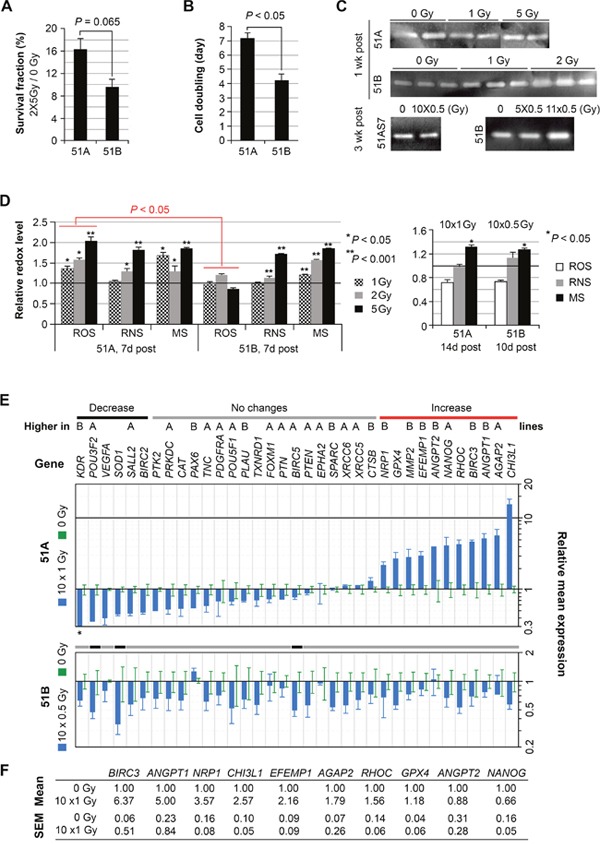
Differential radiation sensitivity and changes of molecular profiles of irradiated GBM subpopulation cells with and without DM, but with common and prolonged increase of mitochondrial superoxide **(A)** survival fraction of cells determined by counting cells not stained by Trypan Blue dye, 1 week after consecutive weekly fractions of the 5-Gy radiation. **(B)** cell doubling time based on the un-irradiated control cells. **(C)** gelatin zymography gels demonstrating persistent increase of MMP2 by 51B, but not 51A, in response to radiation. **(D)** comparison of reactive oxygen (ROS), and nitrogen (RNS) species and mitochondrial superoxide (MS) levels measured by fluorescence activated cell sorting of cells loaded with CMH_2_DCFDA, DAF, and MitoSOX, respectively, 7-14 days after acute and chronic IRs. All values were normalized to un-irradiated controls, which were set to unity. Error bars represent SEM derived from 4 replicates. **(E-F)** comparison of gene expressions, quantified by real-time qRT-PCR, in cells from two independent radiation experiments.

The difference in cellular bioenergetics of 51A, with a higher glycolytic respiration and lower oxidative phosphorylation compared to 51B, and the versatility in switching between two respiration systems upon radiation, suggested that 51A was more radio-resistant. Since 51B cells cannot form a colony either under adherent conditions or in soft agar, we determined the relative radiosensitivity of 51A and 51B by measurements of cell proliferation conducted 1 week after two weekly exposures (5 Gy each), and comparing to corresponding cells (seeded in half amount) without IR. Trypan Blue dye was added to trypsin-EDTA during the process of dissociating cells, followed by adding 10% serum to inhibit cell digestion. The blue-stained cells were considered to be inviable cells from IR as they lacked the ability to exclude Trypan Blue dye, and were excluded in cell counting. When normalized against un-irradiated controls, the surviving fraction of 51A was nearly twice that of 51B (Figure 5A). In this approach to determining the surviving fraction and comparing the two lines of cells, the surviving fraction of the slow-growing line may be underestimated in comparison to that of the fast-growing line. Since it is the slow-growing 51A cells (Figure 5B) that showed a higher surviving fraction in comparison of 51B, 51A cells were more resistant to IR than 51B cells.

We then carried out a study of the radiation effect on molecular changes measured by gelatin zymography and AqRT-PCR, and on redox status by fluorescence activated cell sorting. In our previous study of radiation effects on glioma cells, we found that the extracellular level of MMP2 was upregulated by radiation [5]. Here, again we found that the high-MMP2-expressing cells of 51B had increased MMP2 1-3 weeks following both acute and chronic IR. In contrast, low-MMP2-expressing cells of 51A did not show this effect (Figure 5C).

We compared the redox status of cells in response to IR. As shown in Figure 5D (left panel), after acute radiation at various dosages, ROS levels were unchanged in 51B, while 51A showed a dose-dependent increase. Following 10-days of chronic radiation and 10-14 days recovery, the ROS and RNS levels were not significantly different from that of unirradiated cells (Figure 5E, right panel), however, mitochondrial superoxide (MS) remained significantly elevated in irradiated cells of 51A and 51B, 1-2 weeks post-acute or chronic IR (Figure 5D, right panel).

A fractionated IR was performed using 51A (3 or 5 x10^6^/T75 flasks for control or 1 Gy x 10 days, respectively) and 51B (2 or 7 × 10^5^/T75 flasks for control or 0.5 Gy x 10 days, respectively). RNA samples were extracted 11 days post IR from 4-day cultured cells (1 × 10^5^/well in triplicate in 12-well plates). AqRT-PCR showed only a few (3 of 36) genes in 51B had altered expression (all being downregulated) 11 days after chronic IR (0.5 Gy per day for 10 days), among which, two (*POU3F2* and *SOD1*) were also downregulated in irradiated 51A (Figure 5E). In contrast, chronic IR of 51A at a higher dose (1 Gy per day for 10 days) caused upregulation of six genes (*MMP2, EFEMP1, ANGPT1, ANGPT2, NRP1*, and *BIRC3*) which were highly expressed in 51B (Figure 2B) and functionally characterized to promote angiogenesis. Other radiation-upregulated genes in 51A were consistent with its radio-resistance (*BIRC*3, *GPX4, CHI3L1*, and *NANOG*) and high invasiveness (*AGAP2* and *RHOC*).

A repeat of the IR experiment was performed using 51A with lower seeding density (8 or 12 × 10^5^/T75 flasks for control or 1 Gy x 10 days, respectively). Large numbers of neurospheres were observed during IR treatment. Nine days post IR, cells were plated in a 24-well plate (1 X10^5^/well in triplicate) and RNA samples were extracted one day later. As shown in Figure 5F, AqRT-PCR confirmed that most of the genes (*BIRC3, ANGPT1, NRP1, CHI3L1, EFEMP1, AGAP2*, and *RHOC*) had IR-induced up-regulation. Overall, the data from two independent IR experiments showed a radiation-regulated gene-expression switch of cells with DM to that expressed by cells without DM.

### Prolonged radiation effect on the tumor microenvironment supporting tumor recolonization

The data above showed the differential radiation-resistance and molecular profiles of two subpopulations with and without DM, and that the surviving cells with DM frequently mis-segregated their DM chromosomes to re-populate the population subset of cells lacking DM (see study on 51AS7 in Figure 1D) with a corresponding change in their molecular phenotype. This suggests that cells with DM are largely responsible for tumor recurrence. Hence, we carried out a study to determine the prolonged paracrine effects of irradiated cells with DM on the progeny of cells that survived IR.

**Figure 6:**
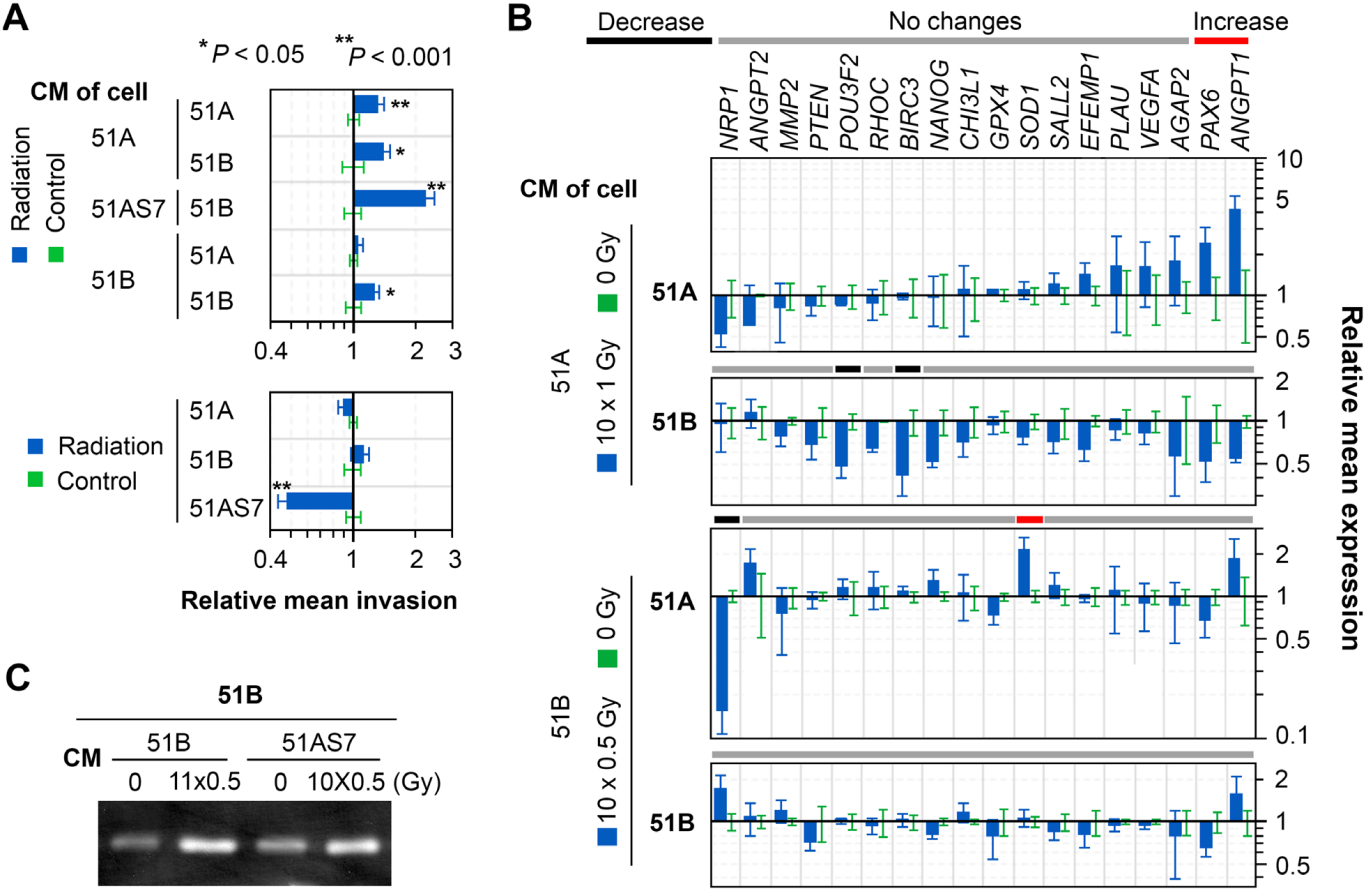
Long-lasting effect from irradiated glioma cells in changing their tumor microenvironment to that essential for tumor angiogenesis and recolonization **(A)** matrigel invasion assay of 51A, 51B, and 51AS7 cells 1 week post radiation (bottom panel) or cultured for 5 days in conditioned medium (CM) of each irradiated line (upper panel). The CM was supplemented with EGF/bFGF/B27 or FBS to culture 51A or 51B, respectively, in monolayer condition. The controls are the cells in experiments without IR. Radiation treatment was as follows: the cells were plated at double the number of the control and exposed to consecutive daily IR of 0.5-Gy for 11 days for 51B or 1-Gy for 10 days for 51A and 51AS7. **(B)** real-time qRT-PCR detected changes in gene expressions 4 days following a 5-day culture in conditioned medium from irradiated cells. **(C)** gelatin zymography assay detected changes in the active level of MMP2 in 2-day conditioned medium of 51B treated by conditioned medium of irradiated cells for 5 days.

We cultured cells from 51A and 51B after chronic radiation in basal medium for 1 and 2 days and used conditioned medium (CM) to culture un-irradiated cells of both types, adding the supplements required by NS and SA-lines (EGF/bFGF/B27 for 51A and 10% fetal bovine serum for 51B) for five days, followed by cell invasion assays and quantification of genes which expressions were regulated by radiation. As shown in Figure 6A (upper panel), radiation altered the components in CM of cells with DM (51A, 51AS7) to promote invasiveness of both cells with or without DM in 51A or 51B, respectively. However, the direct IR of cells failed to promote, or even decreased, cell invasion, likely due to radiation-induced cellular damage (Figure 6A bottom panel).

Two marker genes (*PAX6, ANGPT1*) of 51B were upregulated in 51A due to exposure to CM from irradiated 51A, but not of 51B (Figure 6B). *SOD1* and *NRP1* were respectively up-, and down-regulated in 51A due to exposure to CM from irradiated 51B, not of 51A, which interestingly, were oppositely affected by IR (Figure 5D). IR had both direct and indirect effects on 51B cells to increase extracellular activated MMP2 (Figures 5C and 6C), but not on *MMP2* gene expression (Figures 5E and 6B). These data suggest that MMP2-activating protein(s) in 51B were up-regulated directly by IR and indirectly by paracrine effects from CM derived from irradiated cells.

**Figure 7:**
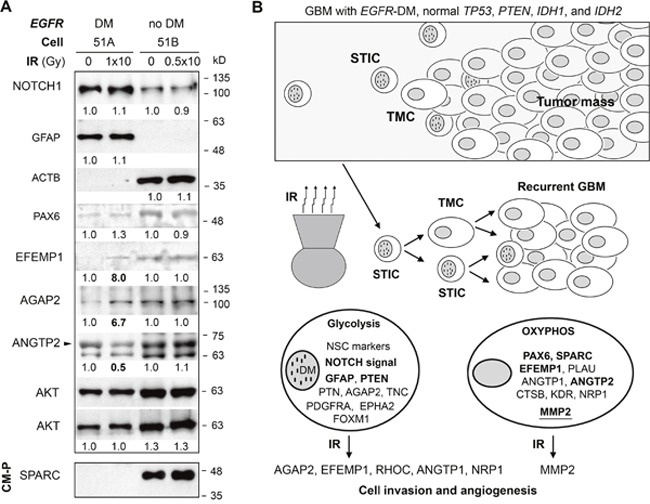
Key molecular and metabolic differences between the DM and no DM subpopulations and their response to IR **(A)** immunoblots of whole-cell lysate or conditioned-medium protein (CM-P, 30 μg for 51A, 3 μg for 51B) of cells with or without IR. AKT was used to control equal protein loading of WCL. Densitometry (carried out using Image J) values are shown below each blot after comparison to un-irradiated control. **(B)** depiction of a model of GBM with *EGFR* amplification and potential mechanism underlying tumor recurrence after radiotherapy. Key cellular respiration machinery and overexpressed genes and hyper activated signaling pathway are shown for two cell subpopulations. Validated gene overexpression at the protein level is shown in bold face. MMP2 is based on enzymatic activity. This model summarizes the overall data showing that GBM cells with *EGFR*-carrying DM are stem-like tumor initiating cells (STICs) that are invasive and radio-resistant. Surviving cells with DM after radiotherapy give rise to cells without DM, which form the tumor mass of a recurrent tumor resembling the original tumor in DM-heterogeneity. IR has a prolonged effect on STIC in promoting its invasiveness and conversion to TMC with a pro-angiogenic tumor microenvironment that supports the growth of TMC.

A repeat of the IR experiment was performed for 51A and 51B with seeding density (3 or 6 × 10^6^/T75 flasks for control or above described IR, respectively). One day post IR, cells were plated in 60 mm dishes (5 × 10^5^/dish), then, 10 days post IR, whole-cell lysates were extracted from the cells. Consistent with RNA-based analysis from the above described IR experiments, protein data shown in Figure 7A confirmed IR-induction of EFEMP1 and AGAP2 in 51A. Immunoblot of 51A showed a reduction of 50% of ANGTP2 by IR, while RNA-based analysis showed contradictory effects of IR on *ANGTP2* RNA (Figure 5E and 5F). Validation of IR-increased expression of *ANGTP1* and *CHI3L1* at the protein level failed due to a lack of specificity of the antibodies when applied against these proteins. Consistent with RNA data showing ne effect of IR on these gene in 51B, none of the IR-regulated protein alterations in 51A were shown in 51B. Results from immunoblotting shown in Figure 7A confirmed much higher levels of NOTCH1 and GFAP in 51A compared to that of 51B, as shown in Figure 2A. Different from prior protein analysis with 51A, this protein assay showed much lower expression of ACTB compared to that of 51B. Reduction in ACTB expression by STIC cells in 51A could be caused by longer culturing in fibronectin-adherent conditions. The higher levels of PAX6 and EFEMP1 in 51B shown in Figure 7A were consistent with prior protein and RNA analyses shown in Figure 2. None of these highly expressed proteins in 51A or 51B were altered by IR.

Using VIVA-20 (30k MWCO PES), equal volumes of 2-day conditioned medium (CM) from 51A and 51B cell cultures (the same amount of cells seeded 1 day post IR and cultured for 9 days), were concentrated 20 fold. Protein measurement showed a nearly 10-fold higher concentration of protein in CM (CM-P) of 51A compared to that of 51B. Immunoblotting of CM-P with antibody against ANGTP1 failed to detect ANGTP1, as shown of WCL, while SPARC antibody detected SPARC made by 51B cells, which were not altered by IR (Figure 7A), confirming the RNA analysis shown in Figure 2C.

## DISCUSSION

Nearly 40% of GBM tumors demonstrate *EGFR* amplification, mostly associated with DM chromosomes. Loss of DM in GBM primary cultures grown under SA conditions (adherent conditions in medium containing serum) has been longly noticed [12]. Under reported neurosphere culture conditions, highly invasive glioma cells with amplification of wild-type *EGFR* have been observed [13]. However, a pair-wise comparison of cell subpopulations with, or without *EGFR* amplification, for their differential role in tumor formation and tumor recurrence post-IR is currently lacking. Using syngeneic GBM cell lines with and without DM, we have shown an invasive phenotype of cells with DM, that are likely a source of tumor recurrence due to their metabolic radioresistance and regenerative plasticity that promotes subsequent restoration of tumor heterogeneity by eliciting subpopulations of cells lacking DM.

Radiation-induced oxidative stress has been shown to alter cell survival parameters through metabolic disruptions that alter the redox state and basal homeostasis of cells. Past work and current results show the capability of acute radiation exposure to elicit an acute and protracted oxidative stress in a variety of neural cell types. Such temporal kinetics of radiation-induced oxidative stress in neural stem and progenitor cells showed similarities with that found in irradiated GBM cells with DM, with a prolonged effect on the elevation of reactive oxygen (ROS) and nitrogen (RNS) species in response to acute radiation. Different alterations on redox status were seen in glioma cells without DM in 51B in which ROS level was stable after acute IR. ROS-regulating gene *GPX4* was upregulated in 51A but unchanged in 51B following IR. This may be related to the increase of ROS level in 51A, whereas there was no change of ROS in 51B in response to IR. The down-regulation of *SOD1* in both 51A and 51B was consistent with their common increase of mitochondrial superoxide (MS) levels after radiation.

It has been observed repeatedly, and for a number of different GBMs, that STIC and TMC subpopulations had different levels of survival after radiation, and that the radio-resistant cell subpopulation was more prone to changes in its respiration machinery [14, 15]. Here we observed again such a phenomenon. After acute IR, metabolism of cells with DM in 51A showed an increase of oxidative phosphorylation and a decrease of glycolysis, while cells without DM in 51B were stable. Furthermore, our findings indicate that the radiation-resistant DM-containing subpopulation possesses the ability to survive and ultimately reestablish the original tumor heterogeneity necessary for GBM virulence. This study has also revealed, for the first time, a paracrine effect of irradiated STIC on un-irradiated, newly formed STIC and TMC, thereby enhancing tumor development/regrowth by promoting cell invasion and angiogenesis. As shown in Figures 5 and 7, radiation downregulated the stem-like cell marker genes while upregulating pro-invasive (AGAP2, EFEMP1, RHOC) and pro-angiogenic (ANGTP1, NRP1) genes/proteins in 51A cells. The increase of extracellular active MMP2 by 51B cells, directly from IR or indirectly from radiated 51A cells, will altered their microenvironment to support growth of recurrent tumors post-IR.

*MMP2*, the key matrix metallopeptidase gene overexpressed in high grade glioma functions in promoting glioma cell invasion [16–19]. However, MMP2 expression level has no prognostic value for GBM (median survival of 0.8 year), while having favorable prognostic values for grade III anaplastic astracytoma (median survival of 3 years), anaplastic oligodendroglioma (median survival 7 years), and grade II oligodendroglioma (median survival 9-10 years) [20]. For the well-known pro-invasive role of MMP2 in cancer, its favorable prognostic effect in low-median grades of glioma may be related to a better survival of glioma with more invasive tumor cells than that with more proliferative tumor cells. GBM is composed of both highly invasive and proliferative tumor cells. By degradation of the extracellular matrix, a high activity level of MMP2 in GBM may support both tumor invasion and growth. Here, in a heterogeneous GBM having cell types with and without EGFR-encoding DM chromosomes, the high MMP2-expressing cell without DM showed high in vitro and *in vivo* cell proliferation, while the low MMP2-expressing cell with DM showed high invasiveness in both *in vitro* and *in vivo* conditions. Radiation further increased the extracellular level of MMP2 by cells without DM. Further study is needed to identify the pro-invasive protein in cells with DM and the contribution of MMP2 to overall tumor growth and angiogenesis in cells without DM.

In these GBM-derived syngeneic primary cultures, the expression of *ANGPT1* was upregulated directly and indirectly by radiation exposure in the STIC with DM subpopulations. *ANGPT1* encodes a secreted glycoprotein that belongs to the angiopoietin family and plays an important role in tumor vascularization as an agonist of angiogenic receptor TEK. In fact, inhibitors of this interaction have been developed as anti-cancer agents [21, 22]. Our finding of *ANGPT1* upregulation as a prolonged response to chronic radiation in the highly infiltrative and radio-resistant STIC (with DM) subpopulation of GBM supports the rationale of using an inhibitor of ANGPT1/TEK following radiotherapy to suppress angiogenesis, and hinder the development of recurrent GBM.

GBM is heterogeneous for cell types, both at the inter-tumoral and intra-tumoral level. That means one GBM is different from another GBM. For better treatment selection, although unfortunately none of the current frontline therapies are effective, GBM can now be classified into four subtypes, based on certain alterations of genes and gene expressions [1]. It is important to characterize intra-tumoral heterogeneity of GBM to understand its recurrence after therapeutic intervention. However, there are limited models thus far to study the mechanisms underlying the maintenance of tumor heterogeneity. Here we provide one for GBM with EGFR amplification, and the approach for further investigation with clinical materials from other GBMs with EGFR amplification.

The purpose of using different culture conditions, here SA and NS culture conditions, in the establishment of the initial primary cultures from clinical GBM samples is to enrich two major tumor cell subpopulations that are dependent on different supplementation in the culture medium. Further subculture of each SA and NS primary culture is to study the ability of different cell subpopulations to restore heterogeneity, i.e., to give rise to the other cell lineage in each subculture. Using a soft agar-mediated clonal sub-culturing approach to study restoration of tumor heterogeneity is based on our observation that one type of cancer cell gives rise to progeny cells of different phenotypes under conditions lacking a threshold cue from its neighboring cells, by mis-segregation of the tumor-specific chromosome (e.g. Chr7) during cell division [6]. In this particular GBM, however, tumor heterogeneity is not re-established via mis-segregation of Chr7, but rather by *EGFR*-containing DM. Whether other GBMs with EGFR amplification engendered by DM have heterogeneity restored by mis-segregation of DMs requires a further investigation using the approach we present in this study.

Chromosome instability is a hallmark of cancer, which has led to the conception of cancer as a chromosomal disease [23, 24]. Chromosome mis-segregation is an inheritable source of diversity in tumor cell populations, which leads to the successful evolution of the cancer and the recurrence of cancer. The GBM we studied, yielding the results presented in this paper, provide the approach for further investigation with clinical materials from other GBMs with *EGFR* amplification. We have shown that all clonal NS-subculture lines with (51AS7) or without (51AS3 and 51AS5) DM showed the same protein profile (PAX6^low^/EGFR-PTEN-NOTCH^high^) as their parental NS-culture line 51A with DM. It appears to be important to this GBM to maintain the tumor subpopulation with EGFR overexpression, manifested via DM or a mechanism of EGFR up-regulation. Whether the high level of EGFR manifested via DM and/or the mechanism of EGFR up-regulation is a marker, and/or causes the observed radio-resistant, invasive and angiogenic phenotype, requires further study.

## MATERIALS AND METHODS

### Ethics statement

The use of fresh GBM tissue and animal in this study has been approved by our institutional review board. Methods of i.c. cell implantation, survival data and sample collection have been described previously [6].

### Syngeneic GBM primary cultures, mutation assays, and lentivirus infection

Fresh GBM tissue (G43) was dissociated enzymatically (0.05% trypsin-EDTA for 30–45 min at 37°C), disrupted mechanically (passing through a glass pipette in DMEM/F12 containing 0.10 mg/ml DNase and 10% serum), and cultured in both collagen-coated (3-4 μg/cm^2^) culture dishes in DMEM/F12 supplemented with 10% fetal bovine serum, designated as serum adherent (SA) culture conditions, and agar (1%)-coated culture dishes in DMEM/F12 supplemented with epidermal growth factor (EGF, 20 ng/ml), basic fibroblast growth factor (FGF, 10 ng/ml), and 5% B27 (Invitrogen, Carlsbad, CA), designated as neural sphere (NS) culture conditions. Monolayer NS culture was achieved by plating single cells dissociated from neural spheres using 0.05% trypsin-EDTA into fibronectin (1 μg/cm^2^)-coated dishes in the same culture medium. Each passage of an NS-culture was achieved by passing through monolayer culture. All cells were maintained at 37°C with 5% CO_2_. Cell line authentication for the 51A, 51AS7, and 51B cell lines was performed by IDEXX BioResearch (Columbia, MO). The short tandem repeat (STR) profile was identical for all three cell lines confirming they originated from the same tumor. The genetic profile established for the cell lines is found in Table 1.

**Table 1:** The genetic profile for 51A, 51AS7, 51B, 51A-GFP, 51B-RFP cell lines derived from a recurrent GBM in a 61-year-old Caucasian female

**Marker**	51A (p5)	51B (p18)	51AS7 (p4)	51A-GFP (p1)	51B-RFP (p3)
**AMEL**	X	X	X	X	X
**CSF1PO**	10, 12	10, 12	10, 12	10, 12	10, 12
**D13S317**	11, 12	11, 12	11, 12	11, 12	11, 12
**D16S539**	11, 13	11, 13	11, 13	11, 13	11, 13
**D18S51**	12, 15	12, 15	12, 15	12, 15	12, 15
**D21S11**	29	29	29	29	29
**D3S1358**	13, 15	13, 15	13, 15	13, 15	13, 15
**D5S818**	10, 11	10, 11	10, 11	10, 11	10, 11
**D7S820**	9	9	9	9	9
**D8S1179**	14	14	14	14	14
**FGA**	20	20	20	20	20
**Penta D**	11, 12	11, 12	11, 12	11, 12	11, 12
**Penta E**	5, 10	5, 10	5, 10	5, 10	5, 10
**TH01**	8, 9	8, 9	8, 9	8, 9	8, 9
**TPOX**	8	8	8	8	8
**vWA**	14	14	14	14	14

For identification of the *TP53* mutation, glioma cDNA samples were PCR amplified using forward primer (5′-GAACAATGGTTCACTGAAGACC-3′) annealing to exon 4, and reverse primer (5′-AGCTCTCGGAACATCTCGAAG-3′) annealing to exon 10 of *TP53*. For identification of the *PTEN* mutation, nested PCR with forward primer (5′- TTCCATCCTGCAGAAGAAGC 3′) annealing to exon 1, and reverse primer (5′- CCATTTTCAGTTTATTCAAGTTTA -3′) annealing to exon 9 of *PTEN*, was based on an initial PCR using glioma cDNA samples with primers of 5′- AAGCAGGCCCAGTCGCTGCA 3′ and 5′- GGTCAGGAAAAGAGAATTGTTC -3′. For identification of the *IDH1* or *IDH2* mutation, glioma DNA samples were PCR amplified by forward primer (5′-CAAGCTATGATTTAGGCATAGAG-3′) annealing to the boundary of intron 3 and exon 4, and reverse primer (5′-CATGCAAAATCACATTATTGCC -3′) annealing to intron 4 of *IDH1*, or by forward primer (5′-GGGACCACTATTATCTCTGTCC-3′) annealing to intron 3, and reverse primer (5′-GGCCTTGTACTGCAGAGACAAG-3′) annealing to intron 4 of *IDH2*. The annealing temperature for PCR was 58°C for all four studied genes, while extension time at 72°C varied, depending on the size of amplicon; 1 min for TP53 (881 bp); 1.5 min for PTEN (1548 in 1^st^ PCR, 1356 bp in 2^nd^ PCR); 0.5 min for *IDH1* (330 bp) and *IDH2* (282 bp). PCR products were purified from single bands cut from an agarose gel, extracted using a Gel DNA Recovery Kit (Zymo, Research, Irvine, CA), and sequenced by Genewiz (South Plainfield, NJ) using forward and/or reverse PCR primers of each gene. Pairwise sequence alignment was performed using BLAST to compare the wild-type sequence with the sequence of PCR samples to identify mutations.

Infectious lentivirus was produced by co-transfection of the lentiviral vector plasmid pGIPZ-Empty and pTRIPZ-Empty (Open Biosystems) with packaging plasmid psPAX2 and envelope plasmid pCMV-VSVG in HEK-293T cells, following the manufacturer's protocol. Two days after co-transfection, culture medium containing lentivirus was filtered (0.45 μm) and applied to glioma cell cultures. The infected glioma cells were selected for 1-2 weeks in culture medium containing puromycin (1.25 μg/ml) and examined under a fluorescence microscope, which verified expression of GFP by 51A infected with pGIPZ-Empty and RFP by 51B infected with pTRIPZ-Empty 1 day after addition of doxycycline (1 μg/ml) to the culture medium.

### Soft agar colony formation

5000 cells were mixed with 1 ml of 0.3% SeaPlaque GTG Agarose (Lonza, NJ) diluted from autoclaved, 5% of agarose in PBS with DMEM/F12 supplemented with 10% fetal bovine serum for 51B or a mitogen supplement as detailed above for 51A, spread onto hardened 0.5% soft agar in the same medium (1 ml per well in four corner wells of a 6-well plate). 1 ml of the same medium was added 2 and 3 weeks later and after an additional 4-5 weeks the colonies were counted under a microscope with a 4X lens.

### Fluorescence *in situ* hybridization (FISH)

FISH was performed on metaphase spreads using Direct Labeled Fluorescent DNA Probe Kits with CEP X/CEPY, EGFR/CEP 7 (Abbott Molecular Inc. Des Plaines, IL) described previously [6]. 250-300 cells per sample were counted under a fluorescent microscope with a 100X lens.

### Immunoblotting, gelatin zymography, and matrigel invasion assays

Primary antibodies for EGFR (rabbit, Cell Signaling), NOTCH1 (rabbit, Cell Signaling), AKT (rabbit, Cell Signaling), PAX6 (rabbit, COVANCE), PTEN (A2B1, mouse, Santa Cruz), NES (mouse, EMD), Gamma Secretase (Ab kit for NCSTN), PSEN1 (rabbit, Cell Signaling), SPARC (mouse, Santa Cruz), AGAP2 (rabbit, Abnova), ANGTP1 (rabbit, Abnova), ANGTP2 (mouse, Abnova), CHIL31 (rabbit, Abnova), and EFEMP1 (rabbit, Abnova) were diluted 1:500 or 1000, and for GFAP (C-19, rabbit, Dako) and ACTB (IgM-specific mouse, Millipore) were diluted 1:10,000 for immunoblotting as described previously [11].

Gelatin zymography and matrigel invasion assays were performed following procedures described previously [11]. For the invasion assay in this study, 5×10^5^ cells in 500 μl DMEM/F12 were loaded in 2-3 replications on Matrigel (1 μg/ml)-coated trans-well in 12-well plates (8μm; Fisher Scientific), and 1 ml DMEM/F12 was added to the bottom chamber. For 51B, addition of a small amount of bovine serum (final 0.05%) in the medium of the bottom chamber was applied to enhance migration of cells in penetration through the transwell-filter after degrading the matrigel, but not to interfere cell-mediated matrix degradation by proteases within serum. Images of invasion were taken 48 hours later and cells were counted.

### Real-time absolute quantitative reverse transcription (AqRT-) PCR and comparative quantitative polymerase chain reaction (CQ-PCR)

Total RNA and genomic DNA samples were extracted from glioma cell cultures and mouse brains carrying glioma xenografts using MiniPrep kits from Zymo Research (Irvine, CA). The cDNA samples were converted from 1-3 μg RNA using Superscript III reverse transcriptase (Invitrogen), diluted more than 20 times with 10 mM Tris. HCl (pH7.5), then used (4 μl/each reaction) in real-time PCR, with SYBR Green I master mix (Roche). Gene expressions were quantified by the AqRT-PCR method [10]. DNA copy number variations were measured by CQ-PCR [25]. AqRT-PCR and CQ-PCR standards and primer mixes were provided by Ziren Rsearch LLC (Irvine, CA).

### Flux analysis on cellular bioenergetics

To measure respiration and mitochondrial function in glioma cells, we employed a Seahorse Bioscience XF24 Extracellular Flux Analyzer (Seahorse Bioscience, North Billerica, MA), and utilized sequential injections of Oligomycin (a complex V inhibitor), FCCP (an uncoupler), and Rotenone (a complex I inhibitor), to dissect components of oxidative phosphorylation by quantification of the oxygen consumption rate (OCR). The level of glycolytic respiration was also measured by the extracellular acidification rate (ECAR), as described previously [4]. Briefly, 51B or 51A cells were respectively seeded into a non-coated or fibronectin-coated 24-well Seahorse XF-24 assay plates at various plating densities and cultured for various days with or without exposure to radiation. On the day of metabolic flux analysis, cells were washed once with unbuffered DMEM/F12 (pH7.4) and incubated at 37°C in the same media in an incubator without CO_2_ for 1 hr. Four baseline measurements of OCAR and ECAR were taken before sequential injection of the following mitochondrial inhibitors (final concentration): oligomycin (1 μg/ml), FCCP (0.3 μM) and rotenone (0.1 μM). Three measurements were taken after addition of each inhibitor. OCR and ECAR values were automatically calculated and recorded by the Seahorse XF-24 software. The basal respiration was calculated by averaging the four measurements of OCR before injection of inhibitors.

### Statistical analysis

Overall mouse survivals for different intracranial gliomas were analyzed using Weibull regression, a parametric survival-regression method, and SAS versions 9.2 and 9.3 (The SAS Institute, Cary, NC). We used the t-test (two tailed) in pairwise comparisons for statistical differences.

## Abbreviations

FISH: fluorescence *in situ* hybridization
Chr7: chromosome 7
DM: double minute chromosomes
IR: irradiation
GBM: glioblastoma multiforme
STIC: stem-like tumor initiating cell
TMC: tumor mass-forming cell
CQ-PCR: comparative quantitative PCR
OCR: oxygen consumption rate
ECAR: extracellular acidification rate.
